# Carboxyamidotriazole Regulates the Function of Salivary Gland Epithelial Cells and B Cells to Alleviate Experimental Sjögren′s Disease in Mice

**DOI:** 10.7150/ijms.111897

**Published:** 2025-04-28

**Authors:** Xiaojuan Zhang, Jingwen Liu, Mei Yang, Juan Li, Lei Zhu

**Affiliations:** 1 Department of Pharmacology, Institute of Basic Medical Sciences, Chinese Academy of Medical Sciences and School of Basic Medicine, Peking Union Medical College, Beijing 100005, China.; 2 Medical Epigenetics Research Center, Chinese Academy of Medical Sciences, Beijing 100005, China.

**Keywords:** carboxyamidotriazole, Sjögren′s disease, NOD/Ltj mice, salivary gland epithelial cells, B cells, cytokines

## Abstract

Sjögren's disease (SjD), a systemic autoimmune disease, suffers from restricted treatment choices. The activation of salivary gland epithelial cells and abnormal auto-reactive B cells, triggering cytokine and autoantibody generation, is key to its immunopathogenesis. Carboxyamidotriazole (CAI) was reported to have anti-inflammatory properties by reducing cytokines, yet its role in SjD was unknown. In this research, we targeted to probe CAI's potential treatment effect on SjD-like NOD/Ltj mice and its mechanism. Utilizing the salivary glands of these mice, we employed HE staining, ELISA, immunohistochemistry and flow cytometry. Findings revealed that CAI augmented salivary secretion, decreased water intake and serum autoantibody levels, suppressed histological alterations and lymphocyte foci, and diminished inflammatory factors such as IL-1β and IL-6. It also blocked IκBα degradation and p65 nuclear translocation. *In vitro*, CAI restrained IL-6 secretion from stimulated SGECs and halted Raji B cells' proliferation at G0/G1 stage. Overall, CAI shows an anti-SjD effect in NOD/Ltj mice, probably by regulating relevant cells and deactivating the NF-κB pathway.

## Introduction

As one of the most common systemic autoimmune diseases, Sjögren′s disease (SjD) is characterized by immune-mediated damage of exocrine glands, especially of salivary and lachrymal glands, which results in glandular dysfunction and the clinical manifestation of xerostomia (dry mouth) and xerophthalmia (dry eyes). Besides, a variety of systemic manifestations may occur, involving virtually any organ system. As a result, a large symptom burden seriously affects the quality of life^1^-^3^.

The etiology and pathogenesis of SjD remain elusive. Anyhow, it is generally accepted that the abnormality of immune cell function is involved in the development of SjD^4^. Especially, the activation of salivary gland epithelial cells (SGECs) and the emergence of aberrant autoreactive B cells, conducting to inflammatory factors and autoantibodies production seem to be a crucial role^5^. SGECs are not only a critical immune target of SjD but also play an essential immune function in the pathogenesis of SjD. Amounts of stimuli, such as virus, bacteria, cytokines or other activators, can cause the hyperactivation of NF-κB in SGECs, producing a variety of cytokines and chemokines to mediate the initiation and persistence of inflammation and autoimmune response in SjD^5^,^6^. Abundant evidence suggests that SGECs can drive the activation, differentiation, and survival of B cells through direct interaction and cytokines (i.e., IL-6 and IL-21) production^7^,^8^. Hyperactivated B cells participate in the immunopathogenesis of SjD as autoantibody secretors, cytokine producers and antigen-presenting cells. Lymphoma, one of the most severe complications of SjD, is also related to B cells activation^9^,^10^. Thus, SGECs, B cells, other inflammatory cells, cytokines and autoantibodies produced by each of them or by each other constitute the unique immune microenvironment, which perpetuate the inflammatory process in exocrine glands and ultimately lead to tissue destruction and dysfunction^4^.

Nowadays, treatment strategies of SjD are largely empirical and offer only relief for the symptoms of dryness. There are no approved therapies shown to alter disease progression or treat the systemic manifestations of disease. Therefore, SjD treatment remains challenging in clinical practice, and the curative agent is urgently needed^11^. Carboxyamidotriazole (CAI) is originally developed as a non-cytotoxic antitumor drug, since its exposure has been demonstrated to inhibit the growth of a wide array of tumour cell types. In recent years, we report that CAI has the considerable anti-inflammatory potency in treating autoimmune diseases^12^-^16^. CAI alleviated inflammation and prevented pannus formation and cartilage erosion in adjuvant-induced rheumatoid arthritis (RA) rat model^12^,^15^. CAI ameliorated intestinal inflammation manifestation in 2,4,6-trinitrobenzene sulfonic acid-induced colitis in rats and dextran sulfate sodium-induced colitis in mice, the animal models that mimic inflammatory bowel diseases (IBD) 13. And the anti-inflammatory effect of CAI is associated with its inhibition of pro-inflammatory cytokines release by blocking NF-κB pathway activation^12^,^14^. However, the efficacy of CAI on SjD remains unknown. In this study we investigated the potential therapeutic effect of CAI in SjD-like NOD/Ltj mice, and explored its mechanism using primary SGECs and Raji B cell line, to emphasize its potential as a new drug for treating SjD.

## Material and methods

### Drugs

CAI was synthesized by the Institute of Materia Medica, Chinese Academy of Medical Sciences (Beijing, China). It was dissolved in polyethylene glycol (PEG) 400 at required concentration for in vivo experiment and in dimethyl sulfoxide (DMSO) as a 40 mM stock solution for in vitro experiment. Total glucosides of paeony (TGP) as a positive control drug was obtained by Liwah pharmaceutical Co., Ltd. (Ningbo, China).

### Animals

Four-week-old female NOD/Ltj, wide-type ICR and C57BL/6 mice were obtained from Beijing HFK Bioscience Co., Ltd. (Beijing, China). They were housed in an air-conditioned room (22 ± 2℃ and 40%-70% humidity), with a controlled 12 h light/dark cycle. Food and water were provided ad libitum. All animal care and experimental procedures were approved by the Institutional Animal Care Use & Welfare Committee of Institute of Basic Medical Sciences, Chinese Academy of Medical Science (the number of ethic approval: ACUC-A02-2021-027).

### Grouping and administration

NOD/Ltj mice were divided into 4 groups (n=20/group) and administrated two doses of CAI (20 and 40 mg/kg, i.g.), TGP (150 mg/kg, i.g.) and vehicle PEG 400 (i.g.) once daily for 16 weeks from 4 weeks old. ICR mice were used as normal control group (n=20). All mice were killed by cervical dislocation when they were 20 weeks old.

### Water intake assay

The drinking bottles of the rearing cage were weighed at a fixed time every day, and the weight difference of the bottles was the total water intake of mice in the cage (each cage contained 5 mice; only mice from the same group were kept in the same cage). The average water intake per group was calculated by dividing total water intake of the group by the number of mice.

### Salivary flow measurement

The saliva flow was measured by wet weight method 17 at the age of 12 weeks and 20 weeks in mice. Briefly, mice were anesthetized by an intraperitoneal injection of 2.4% sodium pentobarbital (5 ml/kg; Sigma, St. Louis, MO, USA) and then intraperitoneally injected with pilocarpine (0.125 mg/kg; Sigma). Four minutes later, the dry and sterile cotton ball was placed into the mouse mouth. After 5 min intervals, the wet cotton ball was taken out. The difference between wet weight and dry weight of the cotton ball was expressed as the mount of saliva collected and standardized as mg for 5 min.

### Measurement of anti-SSA/Ro, anti-SSB/La and IgG antibodies

At the age of 20 weeks, blood samples were collected from the retro-orbital plexus of mice before they were sacrificed. Serum was separated from the blood by centrifuging at 3,000 rpm for 20 min at 4 ℃. The concentrations of anti-SSA/Ro, anti-SSB/La and IgG antibodies in serum were measured by corresponding enzyme-linked immunosorbent assay (ELISA) kits according to the manufacturer's protocol. ELISA kits for mouse anti-SSA/Ro and anti-SSB/La antibodies were purchased from Alpha Diagnostic Intl., Inc. (San Antonio, Texas, USA), and mouse IgG ELISA kit was purchased from Abcam Co. (Cambridge, UK).

### Calculation of salivary glands and spleen indexes

The mice were sacrificed after blood collection. Then, salivary glands (SGs) and spleen were extracted and weighed. Organ indexes were calculated based on the following formulas: SG index (mg/g) = SGs weight (mg)/body weight (g), spleen index (mg/g) = spleen weight (mg)/body weight (g).

### Histological analysis

SGs were fixed in 10% neutral-buffered formalin and embedded in paraffin for hematoxylin and eosin (HE) staining. The severity of lymphocytic infiltration in SGs was estimated using the Chisholm-Mason histological scoring criteria in which a lymphocytic foci was defined as a group of >50 lymphocytes in a random field (4 mm^2^) of the section. The numerical rating scores were as following: 0, no lymphocytic infiltration; 1, slight lymphocytic infiltration; 2, moderate lymphocytic infiltration but less than one lymphocytic foci; 3, one lymphocytic foci; 4, more than one lymphocytic foci^18^.

### Immunohistochemistry (IHC)

The paraffin-embedded SGs sections (4 mm) were deparaffinized and underwent antigen retrieval. After removement of endogenous peroxidase using 3% H_2_O_2_, the sections were blocked in 10% goat serum for 30 min and incubated with the primary anti-p65 mAb (Abcam) or anti-IκBα mAb (Abcam) at 4 ℃ overnight. Then, the sections were incubated with secondary antibody followed by stained with diaminobenzidine (DAB) chromogenic solution. Hematoxylin was used for nuclear counterstaining. For semiquantitative analysis of the immunohistochemical findings, six fields in each section were inspected and graded. Immunohistochemical score was calculated based on the sum of a proportion score (percent of positive-stained cells: 0, none; 1, <30%; 2, 30%-70%; and 3, >70%) and intensity score (0, none; 1, weak; 2, intermediate; and 3, strong). And the numerical rating score were as follows: 0, negative; 2-3, weak positive; 4, positive; 5-6, strong positive^12^.

### SGECs isolation and culture

The isolation and culture of SGECs were based on that used in our previous study^19^. In brief, SG tissues were extracted from C57BL/6 mice which were sacrificed by cervical dislocation, and then digested by collagenase type IV (0.25 g/L; Sigma, St. Louis, MO, USA) at 37°C in a humidified atmosphere of 5% CO_2_ for 3 h. The digested tissues were filtered using a 100 mm filter and the resulting cell suspension was centrifuged at 1,000 rpm for 5 min. After purified by differential attachment method, SGECs were cultured in F-12/DMEM medium containing 2.5% fetal bovine serum (FBS), 10 μg/L epidermal growth factor, 2 mmol/L L-glutamine, 100 U/mL penicillin, and 100 μg/mL streptomycin.

### Raji B cells culture

Human neoplastic B lymphoid cell line Raji was obtained from the Cell Resource Center, Peking Union Medical College (which is part of the National Science and Technology Infrastructure, the National Biomedical Cell-Line Resource, NSTI-BMCR). Raji cells were cultured in RPMI 1640-modified culture medium supplemented with 10% FBS, 100 U/mL penicillin, and 100 μg/mL streptomycin.

### Cell viability, proliferation, and apoptosis assays

Cell counting kit 8 (CCK8) assay was used to evaluate the cell viability of SGECs and the proliferation of Raji cells. In brief, SGECs and Raji cells were plated in 96-well plates at a density of 5×10^3^/well or 5×10^5^/well, respectively, and treated as indicated. CCK-8 solution (10 µL/well; Biosharp, Hefei, China) was added 4 h (for SGECs) or 2 h (for Raji cells) prior to absorbance measurements. The absorbance was measured at 450 nm on a BioTek Synergy H11 microplate spectrophotometer. For the apoptosis assay, Raji cells were seeded in 6 well-plates and treated with DMSO, CAI (5 and 10 μmol/L) for 24 h. The APC Annexin V Apoptosis Detection Kit with PI (Biolegend, San Diego, CA, USA) was used according to the manufacturer's protocol. Briefly, resuspended cells were collected in flow cytometry tubes and stained. Apoptotic cells were analyzed by flow cytometry.

### Cell cycle analysis

Raji cells were seeded in 6 well-plates and treated with DMSO, CAI (5 and 10 μmol/L) for 24h. The Cell Cycle Assay Kit (PI/RNase Staining) (Biorigin, Beijing, China) was used according to the manufacturer's protocol. Briefly, resuspended cells were collected in flow cytometry tubes and stained. Cellular DNA content was analyzed by flow cytometry.

### Cytokine measurement

SG tissues were homogenized in ice-cold tissue protein extraction reagent (ThermoFisher Scientific, Waltham, MA, USA). The homogenate was centrifuged (3,000 rpm for 10 min; 4 ℃), and the supernatants were stored at -80℃. The levels of IL-1β, IL-6, TNF-α and CXCL1/GRO-α in the SG homogenate supernatants were measured by electrochemiluminescence (ECL) kit (Meso Scale Discovery, Rockville, MD, USA) according to the manufacturer's instructions. In addition, IL-6 level in SGECs culture supernatants was determined using IL-6 ELISA kit (ExCell Bio, Shanghai, China) according to the manufacturer's instructions.

### Statistical analysis

All data were reported as mean±SD. All statistical significance was determined by one-way ANOVA followed by Dunnett's test between PEG 400 and other groups using GraphPad Prism version 5.0 software. *P* < 0.05 was considered as a significant difference.

## Results

### CAI alleviates SjD-like manifestation in NOD/Ltj mice

**Figure 1:**
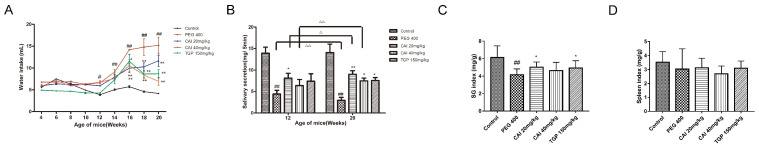
**CAI alleviated SjD-like manifestation in NOD/Ltj model mice. A** The average water intake of mice was measured and calculated during the experiment. **B** The salivary flow of the mice was assessed at the age of 12 weeks and 20 weeks. **C, D** SG index (C) and spleen index (D) of mice were evaluated at the age of 20 weeks. ^#^*P* < 0.05, ^##^*P* < 0.01 compared with the control group; ^*^P < 0.05, ^**^P < 0.01 compared with PEG 400 group. ^△^*P* < 0.05, ^△△^*P* < 0.01 compared with the age of 12 weeks, *n* = 8-12.

It is well known that in SjD, saliva secretion is markedly decreased because of lymphocyte infiltration and subsequent tissue destruction, which leads to the symptom of xerostomia and increase of water intake^1^, ^20^. Therefore, the efficacy of CAI was evaluated by measuring the water intake and saliva secretion of SS-like NOD/Ltj mice. Compared with the normal ICR control mice, the water intake of the vehicle PEG 400-treated NOD/Ltj mice was gradually increased from 12 weeks of age. However, CAI (20 and 40 mg/kg) treatment significantly inhibited the increased water intake in NOD/Ltj mice from 16 weeks of age. Similar effects were observed in NOD/Ltj mice treated with the positive control TGP (Fig. 1A).

We also assessed saliva secretion by determining the saliva flow in mice at 12 weeks and 20 weeks of age. The saliva flow of PEG 400 group was decreased significantly compared to the normal control group from 12 weeks of age when the water intake began to increase, and continued to 20 weeks. Furthermore, the saliva flow at the age of 20 weeks was lower than that at 12 weeks, indicating the progression of salivary glands dysfunction in NOD/Ltj mice. Compared with PEG 400 group, CAI (20 mg/kg)-treated NOD/Ltj mice showed the marked increase in the saliva flow at the age of both 12 and 20 weeks, and the treatment with CAI (40 mg/kg) and TGP increased the amount of saliva flow in 20-week-old mice. It was worth noting that the salivary flow of mice in CAI (20 and 40 mg/kg) group at the age of 20 weeks was higher than that at 12 weeks old (Fig. 1B), suggesting that CAI could improve the significant decrease of salivary flow with age in NOD mice.

The SG and spleen indexes of 20-week-old mice were calculated. As shown in Fig. 1C, treatment with CAI (20 mg/kg) or TGP increased the decreased SG index in NOD/Ltj mice. Additionally, there was no significant difference in spleen index between groups (Fig. 1D). These results suggested that CAI could improve saliva production and dry mouth in SS-like NOD/Ltj mice.

### CAI attenuates lymphocytes infiltration in NOD/Ltj mice

**Figure 2:**
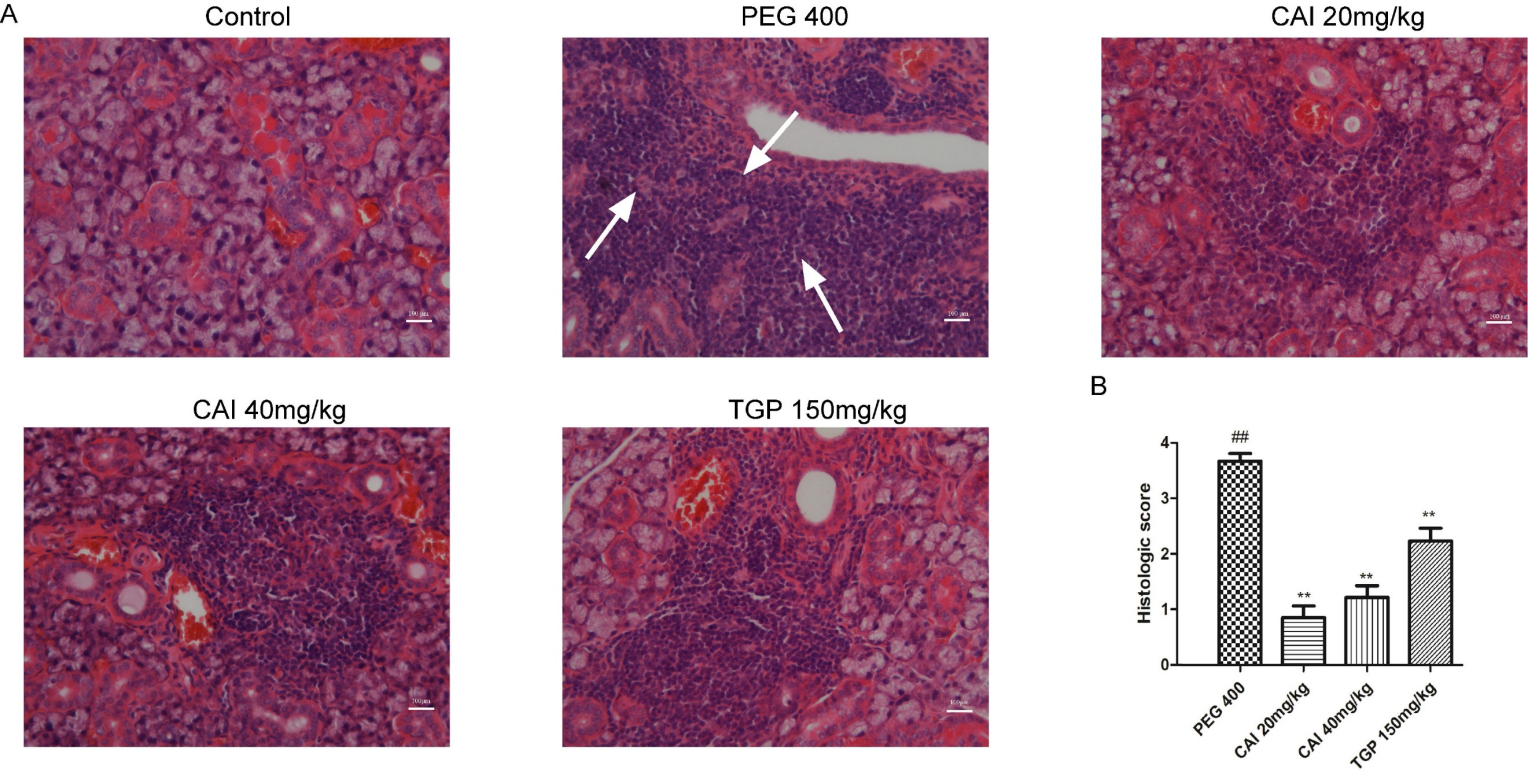
** CAI attenuated lymphocytes infiltration in NOD/Ltj mice. A** Representative HE staining images of SGs from each group. White arrows represent lymphocytic infiltration. Original magnification × 200, bar=100 μm. **B** The severity of gland tissue lesions was assessed for histological scores as defined in Materials and Methods. ^##^*P* < 0.01 compared with the control group; ^**^P < 0.01 compared with PEG 400 group, *n* = 10-14.

HE staining was performed to analyze the histology of acini and lymphocytic foci. As shown in Fig. 2A, the control group showed clear acinus structure without lymphocytic infiltration. However, NOD/Ltj mice developed severe lymphocytic infiltration, and the structure of the acinus in the lymphocyte infiltration was destroyed or disappeared. In comparison, the degrees of lymphocyte infiltration and the destruction of the acinar structure in CAI (20 and 40 mg/kg) and TGP groups were all improved to some extent. Through Chisholm-Mason histology scoring system, the number of lymphocytic foci was quantified. It was found that treatment with CAI and TGP obviously decrease the scores of NOD/Ltj mice (Fig. 2B), indicating that CAI could alleviate lymphocyte infiltration in SS-like NOD/Ltj mice.

### CAI decreases the autoantibodies levels in serum of NOD/Ltj mice

**Figure 3:**
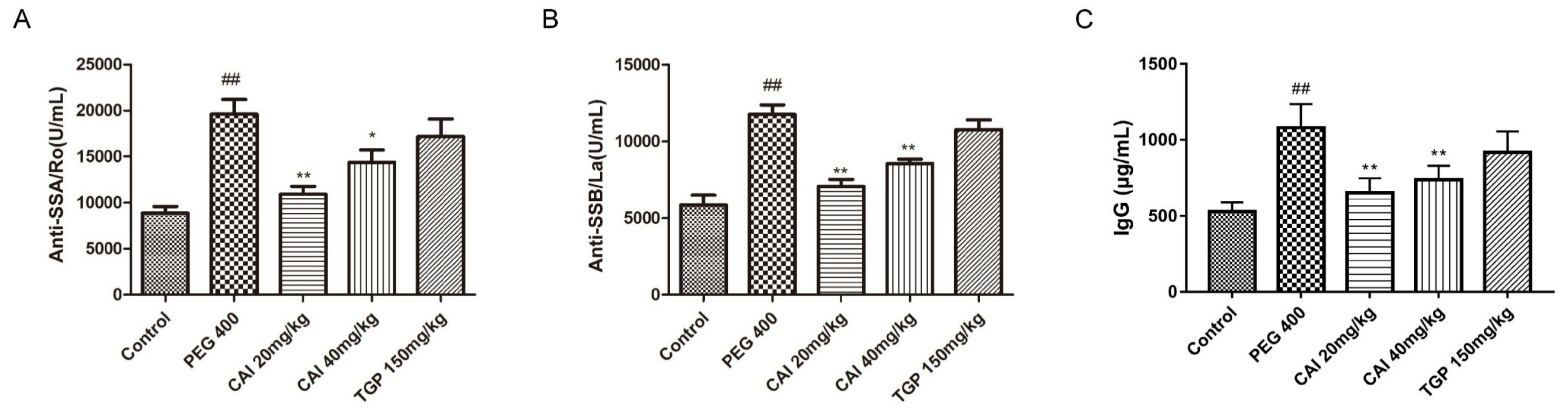
** CAI decreased the levels of SS-related autoantibodies.** Mice were sacrificed at the age of 20 weeks, and serum was collected from the peripheral blood. **A** The level of anti-SSA/Ro level was detected by ELISA kit. **B** The level of anti-SSB/La was detected by ELISA kit. **C** The level of IgG was detected by ELISA kit. ^##^*P* < 0.01 compared with the control group; ^*^P < 0.05, ^**^P < 0.01 compared with PEG 400 group, *n* = 6-8.

The presence of autoantibodies is not only a key feature of SjD, but also an important indicator to support its diagnosis^21^,^22^. Therefore, the levels of several autoantibodies including anti-SSA/Ro, anti-SSB/La and IgG were determined in the serum of mice. The results showed that the anti-SSA/Ro, anti-SSB/La and IgG antibody levels increased significantly in PEG 400 group in comparison with the control group. And there was an obvious decrease of these autoantibodies after CAI (20 and 40 mg/kg) or TGP treatment (Fig. 3).

### CAI reduces the levels of immune-inflammatory cytokines in SGs of NOD/Ltj mice

**Figure 4:**
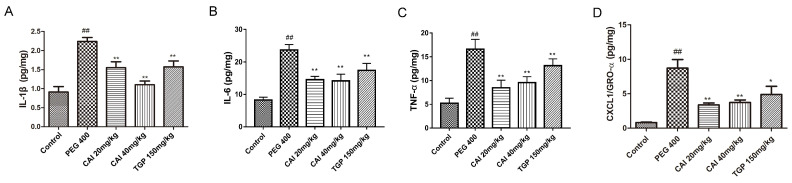
** CAI reduced the concentrations of immune-inflammatory cytokine in NOD/Ltj mice.** Mice were sacrificed at the age of 20 weeks, SGs were extracted and homogenized. The levels of IL-1β (**A**), IL-6 (**B**), TNF-α (**C**), CXCL1/GRO-α (**D**) in homogenates were analyzed by ECL kit. ^##^*P* < 0.01 compared with the control group; ^*^P < 0.05, ^**^P < 0.01 compared with the PEG 400 group, *n* = 5-6.

Since cytokine network dysfunction play an important role in mediating the persistence of immune and inflammatory processes in the exocrine glands of SjD^23^ the effects of CAI on them in NOD/Ltj mice were measured. PEG 400-treated NOD/Ltj mice had higher levels of IL-1β, IL-6, TNF-α and CXCL1/GRO-α in SGs than normal controls, which were found to be markedly decreased by treatment with CAI (20 and 40 mg/kg) or TGP (Fig. 4).

### CAI down-regulates the activation of NF-κB pathway in SGs of NOD/Ltj mice

**Figure 5:**
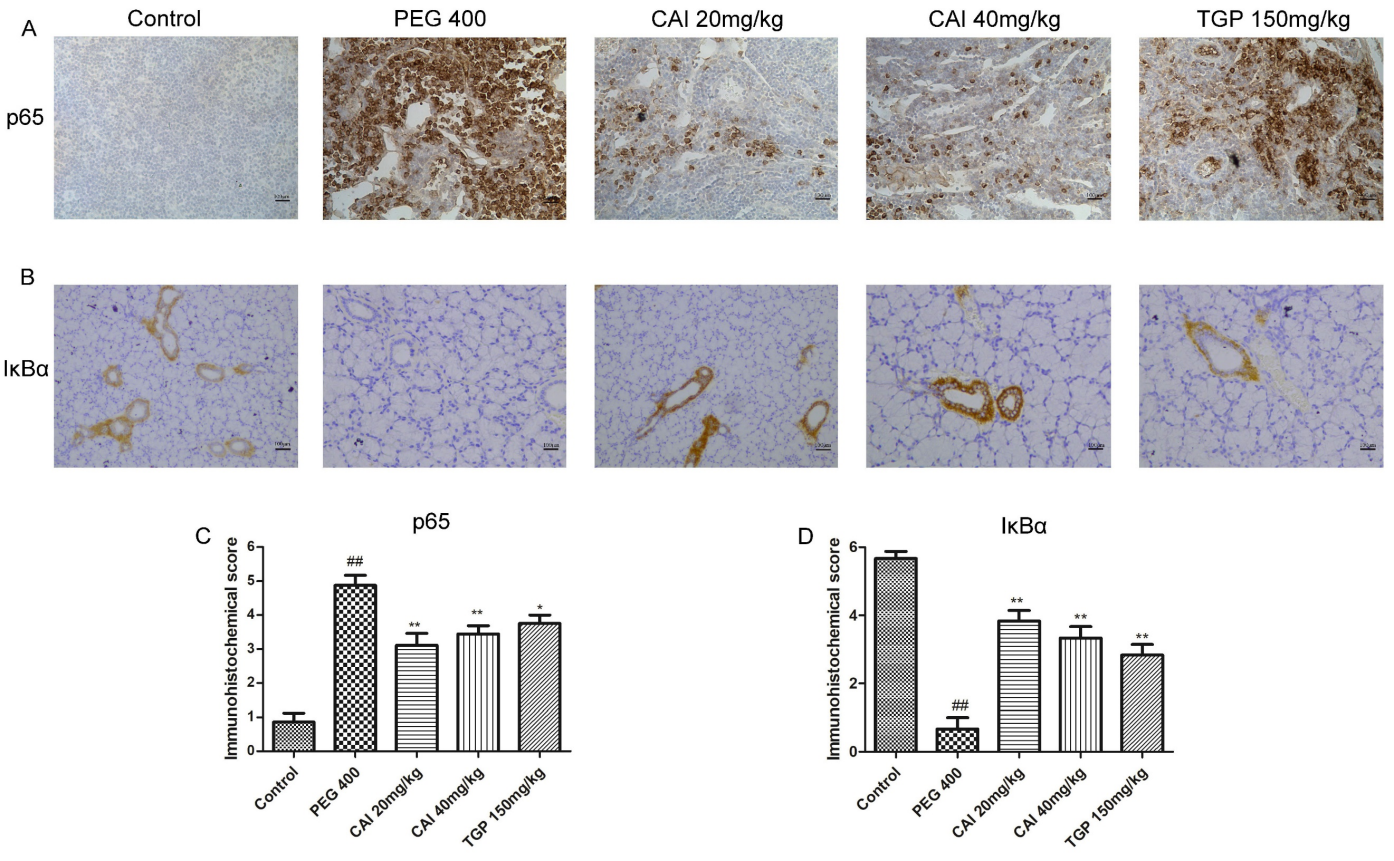
** CAI inhibited NF-κB activation in NOD/Ltj mice. A** Representative IHC staining of the NF-κB p65 subunit in SGs of the indicated groups. **B** Representative IHC staining of IκB in SGs of the indicated groups. Original magnification×200, bar=100 μm. **C, D** Semiquantitative analysis of the immunohistochemical results as defined in Materials and Methods. ^##^*P* < 0.01 compared with the control group; ^*^P < 0.05, ^**^P < 0.01 compared with PEG 400 group, *n* = 7-9.

NF-κB is a family of transcriptional factors that regulates many cellular processes, notably the immune response and inflammation, influencing the transcription of a broad array of pro-inflammatory cytokines^24^. We examined the expression levels of NF-κB signaling-related proteins to evaluated the effects of CAI on this pathway. IHC results showed that the NF-κB p65 subunit expression in the SGs of the control group was in a low level and mainly located in the cytoplasm. The expression of NF-κB p65 in PEG 400 group was much higher than that of the control group and particularly located in the cell nucleus. Treatment with CAI (20 and 40 mg/kg) or TGP could significantly decrease p65 staining both in the cell nucleus and in the cytoplasm (Fig. 5 A and C).

Given the release and nuclear translocation of NF-κB p65 subunit is regulated by the degradation of the inhibitory protein IΚB α, we further determined whether CAI affect IΚB α level in NOD/Ltj mice. Through IHC analysis, we found that the expression level of IΚB α were obviously reduced in SGs of PEG 400 group, compared with the control group. Treatment with CAI (20 and 40 mg/kg), as well as TGP, enhanced the expression of IκBα (Fig. 5 B and D). These results suggested that CAI could inhibit NF-κB activation in NOD/Ltj mice.

### CAI modulates the function of SGECs to secret cytokines

**Figure 6:**
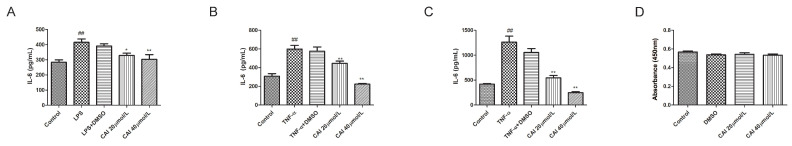
** CAI downregulated stimulus-induced IL-6 secretion in SGECs**. **A** SGECs were incubated with vehicle (0.1% DMSO) or CAI (20 and 40 μmol/L) together with LPS (1 µg/mL) for 24 h. IL-6 level in the supernants were detected by ELISA kit. **B, C** SGECs were incubated with vehicle (0.1% DMSO) or CAI (20 and 40 μmol/L) together with TNF-α (100 ng/mL) for 24 h (B) and 48 h (C), respectively. IL-6 level in the supernants was detected by ELISA kit. **D** Effect of CAI on SGECs viability was evaluated using a CCK-8 assay. ^##^*P* < 0.01 compared with the control group; ^*^P < 0.05, ^**^P < 0.01 compared with stimulus-alone group, *n* = 4-6.

Accumulating evidence has pointed that SGECs are the major players in the pathogenesis of SjD, since they represent the target of the autoimmune process, but also the triggers of the immune activation^5^. Under various stimulus conditions, SGECs produce a variety of cytokines, especially IL-6, which is closely related to B cells hyperactivation in SjD^23^,^25^. Therefore, we isolated and cultured SGECs, and found that lipopolysaccharide (LPS) stimulation increased the level of IL-6 in the supernatant compared with controls, while CAI (20 and 40 μmol/L) inhibited LPS-induced IL-6 secretion (Fig. 6A). TNF-α, a key contributor to SjD pathogenesis, was further used to stimulate SGECs. The results showed that the stimulation of TNF-α for 24 or 48 h upregulated the secretion level of IL-6, which was attenuated by CAI at 20 and 40 μmol/L (Fig. 6B and C). Additionally, CAI did not affect SGECs viability at the concentrations tested compared with controls, which indicated that the inhibitory effect CAI on IL-6 secretion was not due to cytotoxicity (Fig. 6D).

### CAI inhibits B cells proliferation via inducing cell cycle arrest

**Figure 7:**
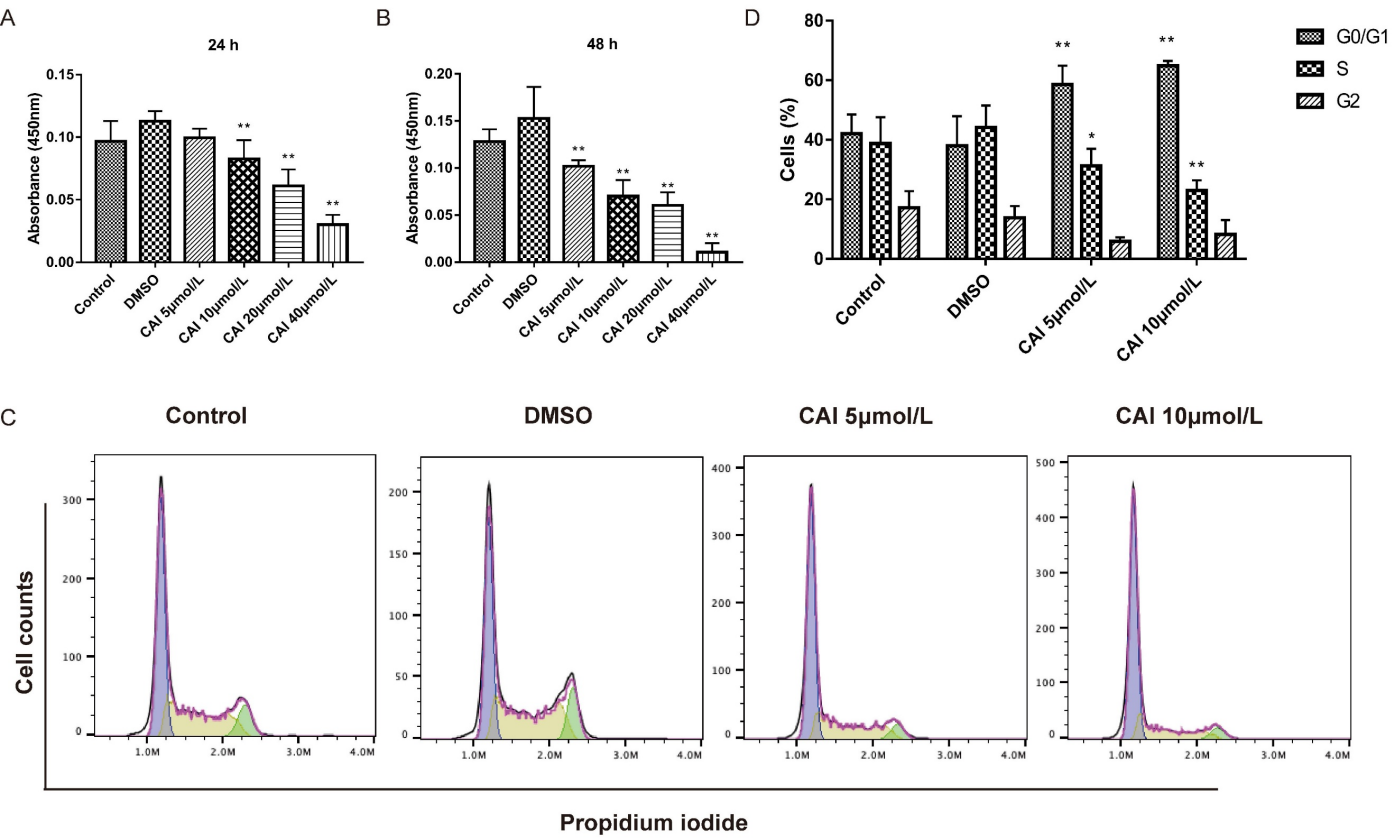
**CAI inhibited B cells proliferation via G0/G1 cell cycle arrest. A, B** Raji cells were incubated with vehicle (0.1% DMSO) or CAI (5, 10, 20 and 40 μmol/L) for 24 h (A) and 48 h (B). The effect of CAI on cells proliferation was measured using a CCK-8 assay. **C** Raji cells were incubated with vehicle (0.1% DMSO) or low concentration of CAI (5 and 10 μmol/L) for 24 h. The effect of CAI on cell cycle progression was tested by flow cytometry-based PI/RNase staining. The representative images from each group were shown. **D** Statistics analysis of flow cytometry. ^*^P < 0.05, ^**^P < 0.01 compared with DMSO group, *n* = 3.

**Figure 8:**
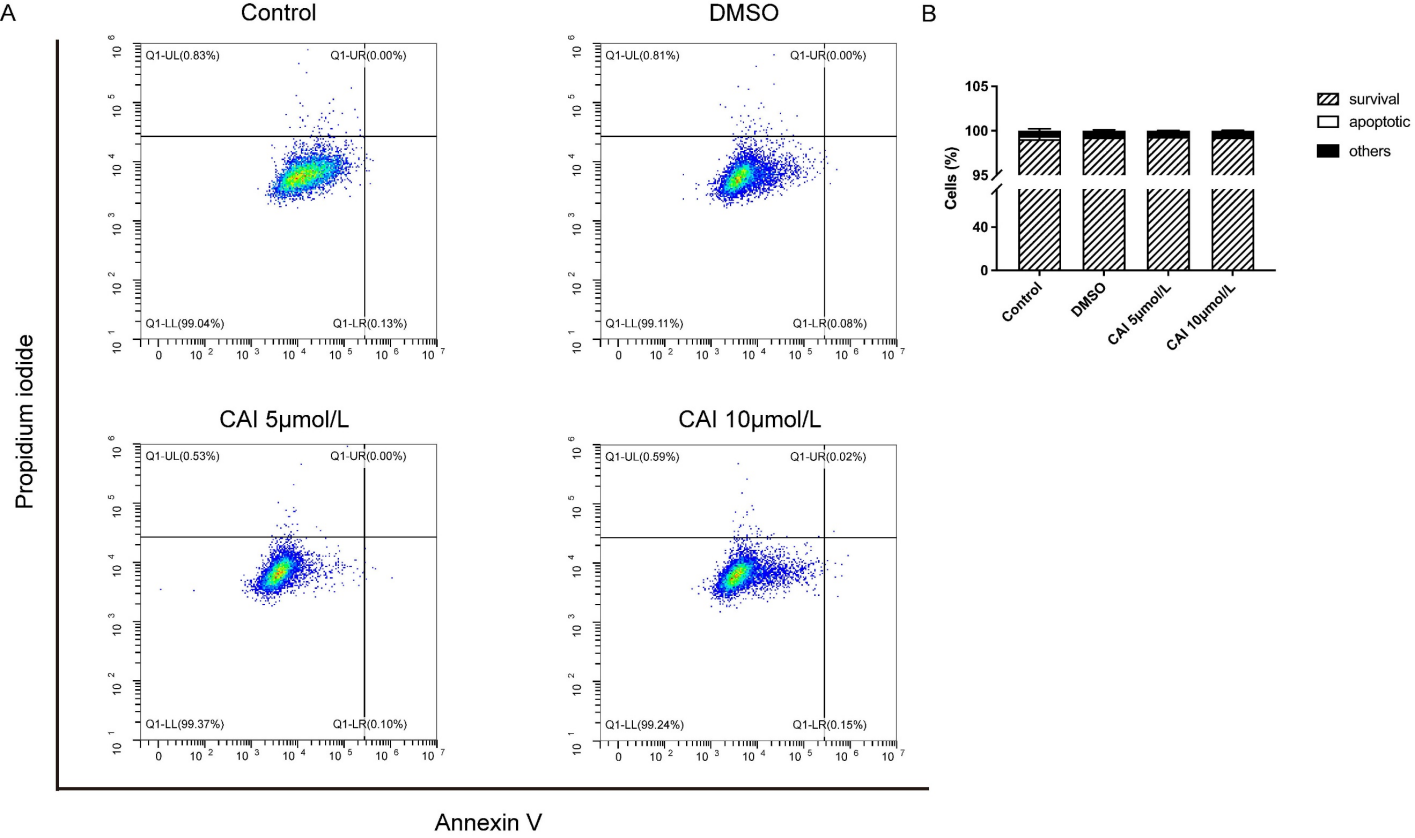
**CAI did not induce apoptosis in Raji cells. A** Raji cells were incubated with vehicle (0.1% DMSO) or low concentration of CAI (5 and 10 μmol/L) for 24 h. The effect of CAI on cell cycle progression was tested by flow cytometry-based Annexin V/PI staining. The representative images from each group were shown. **B** Statistics analysis of flow cytometry, *n* = 4-5.

CAI-treated NOD/Ltj mice produced significantly lower autoantibodies levels than untreated NOD/Ltj mice as shown in Fig. 3, indicating that CAI might inhibit B cells activation in NOD/Ltj mice. As Raji cells have been widely used as *in vitro* model for studying B cells hyperactivation in autoimmune disorders^26^, it was chosen to explore the effect of CAI on B cells in this study. The results showed that CAI treatment for 24 h and 48 h could inhibit the proliferation of Raji cells in a concentration-dependent manner (Fig. 7 A and B). Flow cytometry-based PI/RNase staining results indicated that Raji cells exhibited markedly augmented proportions of cells in G0/G1 phase and lowered proportions of cells in S phase even after treatment with the low concentration of CAI (5 and 10 μmol/L) for 24 h, as compared with the control group (Fig. 7 C and D). However, Annexin V/PI staining assay revealed that the apoptotic ratio of Raji cells was not significantly changed between groups (Fig. 8). These results demonstrated that CAI could inhibit B cells proliferation via G0/G1 cell cycle arrest, which might explain its capacity in diminishing SjD-related autoantibodies.

## Discussion

Animal models have served as useful tools for studying SjD disease pathogenesis and management. To date, various mice models resembling the clinical SjD have been established. One of them is the mouse strain NOD/Ltj, a spontaneous SjD animal model, which has been derived from a cataract-prone strain of outbred ICR mice. However, NOD/Ltj mice are not cataract-prone. Rather, they spontaneously develop the typical SjD-like characteristics, such as lymphocyte infiltration in the salivary and lacrimal glands followed by decreased exocrine function of these glands, that is more similar to patients with SjD than has been seen in any other animal model. Furthermore, the autoantibody signature in NOD model resembles human SjD patients with the presence of anti-SSA/Ro and anti-SSB/La autoantibodies. Therefore, NOD/Ltj mice are recognized to be the most appropriate experimental SjD model to elucidate the pathogenic mechanisms of SjD and to find more effective therapeutic drugs^27^-^29^. In this study, we also observed these SjD-like clinical, immunological and histological features in NOD/Ltj mice. However, the treatment of CAI restored saliva secretion, decreased water intake and increased SG index in treated NOD/Ltj mice. At the histological level, CAI reduced the number of infiltrating lymphocytes and lightened tissue destruction in salivary glands of treated mice. It is well known that in SjD, autoantibodies in the salivary glands induce abnormal immune responses, which together with the infiltrating inflammatory cells destroy normal salivary structure followed by secretory dysfunction. 5 Our results showed that the increased autoantibody levels including anti-SSA/Ro, anti-SSB/La and IgG in NOD/Ltj mice were significantly inhibited after CAI treatment. All of the above findings indicated that CAI could effectively alleviate SjD-like responses in NOD/Ltj mice and, therefore, might be a potentially effective drug for treating SjD.

Several dysfunctional signaling pathways are involved in the immunopathogenesis of SjD. NF-κB is the most important one, which is supported by the observations that drugs through the NF-κB pathway, such as iguratimod and the proteasome inhibitor bortezomib (reported to inhibit activation and nuclear translocation of NF-κB), although has not been approved for the treatment of SjD, clinical studies have shown that they can help improve the clinical symptoms of SjD patients^5^, ^30^-^33^. NF-κB, usually a dimer of p65 and p50 subunits, resides in the cytosol in an inactive state bound to the inhibitory protein IκBα. In response to proinflammatory signals, IκBα can be rapidly degraded, enabling the release and translocation of NF-κB dimer into the nucleus to bind κB enhancer elements of target genes^34^. IκB-defective mice were reported to spontaneously develop SjD-like manifestation^35^. In SjD SGECs and peripheral blood monocytes, the expression level of IκBα was significantly lower than that in healthy controls^36^, ^37^. And lip biopsies' minor salivary glands of SjD patients showed nuclear translocation of NF-κB in focal lymphocytes of infiltrates and in the acini epithelial cells adjacent to the infiltrates^38^. A large number of researches shed light on the importance of the pro-inflammatory cytokines produced by NF-κB overactivation in SjD. They are the important contributors to the inflammatory response during the development of SjD^23^. In particular, IL-6 is critical to plasma cell differentiation and B cell activation in SjD^39^. TNF-α disrupts tight junction structure of epithelial cells resulting in reduced secretory function and gland atrophy^40^. Inflammatory conditions are, in fact, almost always characterized by increased metalloproteinases activities, which lead to destruction of normal tissue architecture. It has been reported that TNF-α and IL-1β can cause glandular structural destruction by inducing matrix metalloproteinases 2^41^. In addition, CXCL1/GRO-α, a member of the CXC chemokine family, not only participates in the inflammatory processes in SjD, but it also^42^ boosts metalloproteinase-17 (ADAM17) activation in SGECs. Consequently, cytokines blockade may be an effective strategy for SjD treatment. However, the trial data for the use of biologics targeting cytokines in SjD has been relatively disappointing. One important reason is that interactions between several interconnected networks of cytokines promote the chronic inflammation in SjD, therefore, inhibition of the cytokine network might be more effective than targeting a one and single cytokine^43^. In this study, the elevated levels of the cytokines including IL-1β, IL-6, TNF-α and CXCL1/GRO-α in SGs of NOD/Ltj mice were markedly suppressed by CAI treatment, possibly due to its inactivation of NF-κB pathway by inhibiting IκBα degradation and p65 nuclear translocation.

The peri-epithelial lymphocytic infiltration of affected exocrine glands and hyperactivity of B cells are the two major characteristics implicated in the autoimmune nature of SjD. SGECs are considered pivotal players in the pathogenesis, designating the disease as an “autoimmune epithelitis” 44. Indeed, SGECs play a double role in SjD, because they represent the target of the autoimmune process, but also the triggers of the immune activation. SGECs are important sources of SjD autoantigens SSA/Ro and SSB/La. After stimulation, SGECs can secrete pro-inflammatory factors that cause infiltration by immune cells and initiation and propagation of immune response. 5 Our studies confirmed that LPS and TNF-α could stimulate SGECs to secrete high level of IL-6, which was significant inhibited by CAI treatment. With the progression of SGECs activation and disease, new immune cells appear in SGs, such as T cells and B cells. Although T cell dysfunctions were initially pointed to play a role in SjD development. Nowadays, accumulating evidence have indicated that the roles of B cells are more critical than T cells. T cells are usually found in mild lesions, whereas it is B cells in the advanced ones, hyperactivity of which results in the production of excessive autoantibodies and cytokines^5^,^9^,^10^. Autoantibodies specific for SjD can develop long before symptoms emerge, highlighting a key early role for B cells in SjD^45^. Therefore, drugs targeting B cells has become an important strategy for alleviating SjD symptoms. In this study, the results showed that CAI could suppress B cells proliferation via G0/G1 cell cycle arrest, which might be a key mechanism for CAI to reduce serum autoantibodies and relieve SjD-like manifestation in NOD/Ltj mice.

In conclusion, the occurrence and development of SjD is a complex process involving interactions between networks of cytokines, SGECs, and B cells. Therefore, targeting these cells and cytokines may be the promising strategy for the treatment of SjD. *In vivo* and *in vitro* assays in this study demonstrate that CAI may be a potent therapeutic agent for SjD, and we hypothesize that CAI plays a key role in inhibiting SjD immunopathogenesis by regulating the function of SGECs and B cells, as well as reducing the production of cytokines via NF-κB pathway inactivation.

## Abbreviations

CAI: carboxyamidotriazole
DMSO: dimethyl sulfoxide
DAB: diaminobenzidine
ELISA: enzyme-linked immunosorbent assay
FBS: fetal bovine serum
HE: hematoxylin and eosin
IHC: Immunohistochemistry
IBD: inflammatory bowel diseases
LPS: lipopolysaccharide
PEG: polyethylene glycol
SjD: Sjögren′s disease
RA: rheumatoid arthritis
SGECs: salivary gland epithelial cells
SGs: salivary glands
TGP: total glucosides of paeony
